# Antimicrobial and mechanical assessment of cellulose-based thermoformable material for invisible dental braces with natural essential oils protecting from biofilm formation

**DOI:** 10.1038/s41598-023-39320-1

**Published:** 2023-08-18

**Authors:** Monika Astasov-Frauenhoffer, Livia Göldi, Nadja Rohr, Sarah Worreth, Elise Dard, Selina Hünerfauth, Tino Töpper, Jonas Zurflüh, Olivier Braissant

**Affiliations:** 1 Department Research, University Center for Dental Medicine Basel UZB, University of Basel, Mattenstrasse 40, Basel, Switzerland; 2 Department of Biomedical Engineering (DBE), Center of Biomechanics and Biocalorimetry, University of Basel, Allschwil, Switzerland; 3 Bottmedical AG Technologiepark Basel, Hochbergerstrasse 60C, 4057 Basel, Switzerland; 4 Department Chemie, University of Basel, Mattenstrasse 24a, Basel, Switzerland

**Keywords:** Dentistry, Microbiology

## Abstract

Controlling biofilm formation in the oral cavity during orthodontic treatments is crucial. Therefore, antimicrobial surfaces for invisible dental appliances are of interest to both therapists and patients. Here we present a cellulose-based thermoformable material used for invisible braces that can be loaded with essential oils (EOs) having antibacterial and antifungal properties. We hypothesize that this material can absorb and release EOs, thus providing an antimicrobial effect without compromising the safety and mechanical properties necessary for dental invisible braces. Conventional microbiology and isothermal microcalorimetry analyses revealed that the thermoformable material loaded with essential oils significantly delayed the biofilm formation of oral streptococci (*S. mutans* and *S. mitis*) under static conditions (*p* < 0.05) and while simulating saliva flow (*p* < 0.05). In addition, cytotoxicity tests (ISO 10993-5), revealed that the loaded material is well tolerated by human gingival fibroblasts. Finally, the loading with antibacterial agents did not significantly alter the mechanical properties and stability of the material (initial force (*p* = 0.916); initial stress (*p* = 0.465)). Compared to gold-standard clear aligner materials, this material offers a reliable transmission of forces for orthodontic treatments. Moreover, this approach exhibits the potential for acting as an oral drug delivery platform for multiple compounds.

## Introduction

Oral health is often taken for granted, however, oral inflammations remain common and may lead to other illnesses including heart disease, diabetes, and neurological disorders like Alzheimer’s^1,2^. Good oral health is associated with good overall health and could even be linked to reduced risk of severe virus infections like COVID-19^3^. Increasing worldwide expenses on cavity treatments, gingival inflammation, or peri-implant disease cause tremendous financial burdens for households and insurance^4^. In addition, with rising numbers of orthodontic treatments, oral care is of even greater importance. As a result, the interest in antimicrobial surfaces for devices such as orthodontic brackets, implants, or invisible dental appliances is currently rising in the oral care community. A study recently showed that after 6 months, 10% of clear aligner patients and 13.3% of removable positioner patients were at risk of caries development due to aligner colonization by *Streptococcus mutans*. This proportion increased to ca 40% in patients with fixed multibrackets appliance. Thus, antimicrobial dental material would be especially beneficial for patients^5^.

To obtain such antimicrobial material, essential oils (EOs) could be particularly useful. EOs are liquid and volatile substances extracted from plants. Many of these components interact with the cell membrane of bacteria due to their hydrophobic nature, making the cell more permeable and potentially leading to cell death. EOs have been proven to have antifungal, antibacterial, antiviral, and insecticidal properties^6–9^. In addition, EOs show a low-level of antimicrobial resistance and a broad spectrum of antimicrobial activity^10,11^. Among the essential oils, cinnamon has an important role because of its antioxidant, anti-inflammatory, antidiabetic, antimicrobial, antitumoral, and lipid-lowering properties. The main bioactive molecule in cinnamon oil is cinnamaldehyde, which has been recognized as safe and nontoxic by the FDA (21 CFR182.6)^8^. With respect to dental applications, cinnamaldehyde has shown low cytotoxicity against fibroblast cell^12^, and neither cinnamon oil nor cinnamaldehyde are considered an intraoral allergen as only rare cases of allergic contact dermatitis have been described. Thus, it is often used as a flavor for various kinds of toothpaste, where cinnamon oil and cinnamaldehyde have additionally shown antimicrobial effects^13,14^. Cinnamaldehyde has not been shown to have an impact on the cold pain threshold; however, it has been demonstrated to lower the mechanical pain threshold^15^. Cinnamaldehyde and cinnamon oils are effective against the early colonizer and caries-causing bacteria *S. mutans*^12,13,16–18^, *Porphyromonas gingivalis*^19^ causing periodontal diseases and *Candida* species potentially leading to dental-induced stomatitis^20,21^. Furthermore, cinnamon extract in a mouthwash showed a decrease in plaque and gingival scores^22^. Finally, cinnamon oils and cinnamaldehyde have good compatibility with other antimicrobials such as eugenol and organic acids resulting in additive or synergistic antibacterial effects^23,24^.

A recent study demonstrated that it is possible to load thermoformable cellulose-based polymers used for invisible braces with cinnamaldehyde^25^. Impregnating these polymer compounds demonstrated a pronounced antimicrobial activity of the material against *Staphylococcus epidermidis* and a weaker but still significant efficacy against oral streptococci^25^. As invisible braces must be worn extensively during the night and even through the day with only minimal removal periods for meals and cleaning, decreasing the risk of biofilm formation and subsequent damage to tooth substance and soft tissue is undoubtedly a valuable preventive strategy.

As EOs are known to have synergistic antimicrobial effects when used with cinnamaldehyde, the possibility of impregnating cellulose-based material with a combination of EO extracts is expected to improve efficacy against oral streptococci. Thus, our study investigated the effect of loading invisible braces made of biopolymers with five bioactive molecules from essential oils. They have been selected based on screening 20 essential oils for their synergistic antimicrobial efficacy against oral streptococci (*S. mutans* and *S. mitis)*. Finally, safety towards human gingival fibroblast and mechanical stability have been analyzed as a prerequisite for using invisible braces in the abovementioned applications. Our study hypothesizes that thermoformable cellulose-based polymers can absorb and release EOs thus providing an antimicrobial effect while keeping necessary safety and mechanical properties comparable to other dental brace material (such as Zendura) or other plastic reference material (such as Thermanox).

## Results

### Bioactive molecules loading and release kinetics based on Gas Chromatography coupled with Mass Spectrometry Analysis (GCMS)

**Figure 1:**
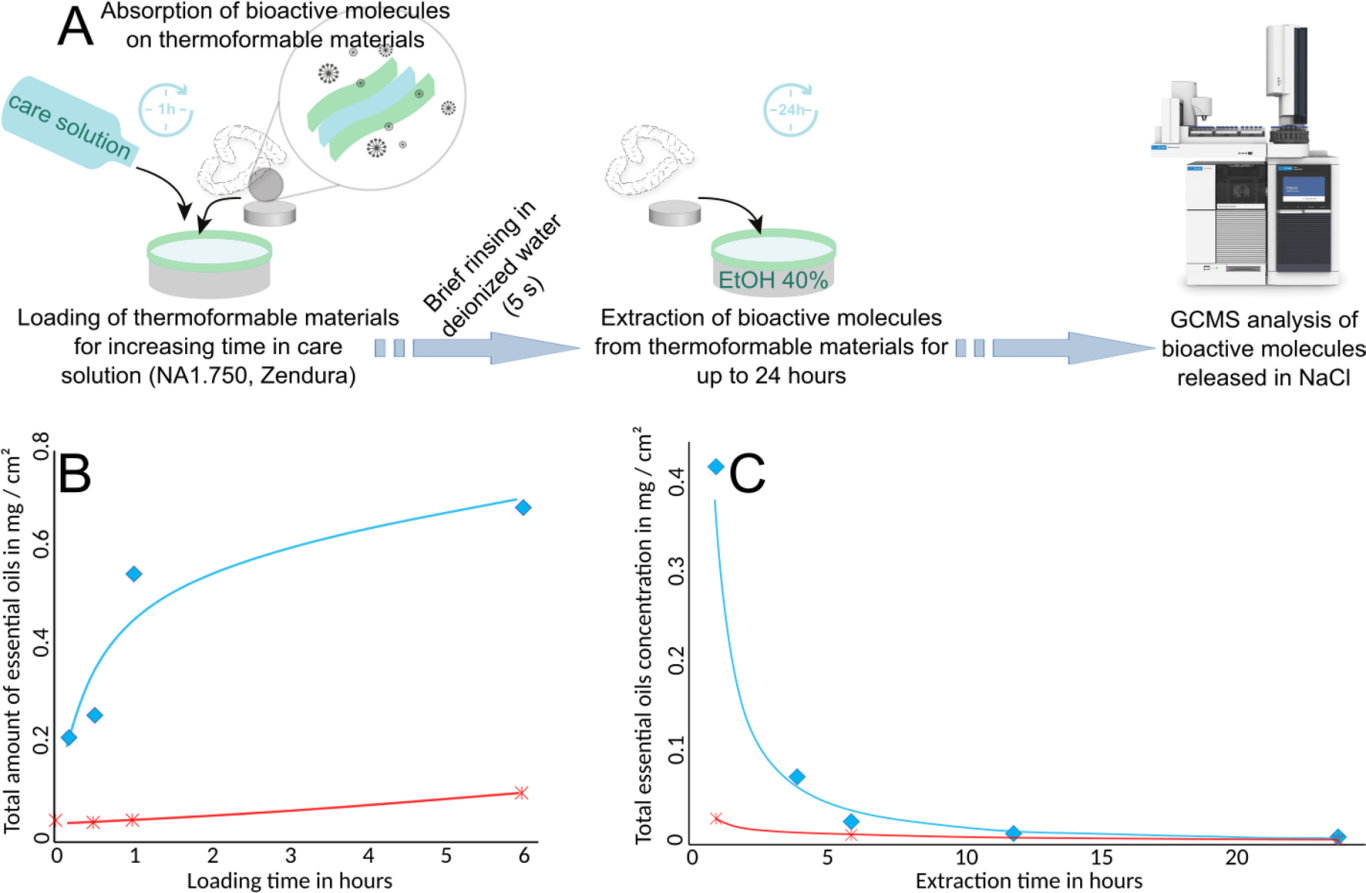
(**A**) Simplified sketch showing the measurement procedure for assessing the loading of thermoformable material with EOs as well as the release kinetic of those EOs when the material is placed in 40% ethanol-solution (see details in the materials and methods section). (**B**) Loading kinetics: Each dot represents the sum of all single EO concentrations absorbed after a certain loading period (blue diamond: NA1.750, red crosses: Zendura). **C)** Release kinetics: Each dot represents the sum of all single essential oil concentrations released after a certain release period (blue diamond: NA1.750, red crosses: Zendura).

GCMS measurements (Fig. 1A) allowed an accurate determination of the amount of bioactive molecules loaded (Fig. 1B) and subsequently released (Fig. 1C) in the extraction solution. The loading kinetic of absorbed bioactive molecules in the thermoformable material (NA1.750) followed a roughly logarithmic pattern. Over time, a concentration of 0.544 mg cm^−2^ of bioactive molecules could be loaded in the NA1.750 after 1 h. After the initial rapid increase in concentration, the amount of bioactive molecules continued increasing up to 0.68 mg cm^−2^ after 6 h however at a slower rate. The individual amounts loaded in the material after 1 h for each compound were quantified as follows: cinnamaldehyde 0.254 mg cm^−2^, methyl-salicylate 0.180 mg cm^−2^, trans-anethole 0.068 mg cm^−2^, eucalyptol 0.027 mg cm^−2^ and limonene 0.015 mg cm^−2^ emphasizing different loading kinetics for the different molecules. For Zendura, used as a marketed reference sample the concentration of bioactive molecule remained at very low concentrations close to the baseline of the resolution limit (i.e., 0.05 mg cm^−2^ after a 6 h loading period).

The measurement of molecule release from the thermoformable material NA1.750 and Zendura loaded for 1 h showed an exponential decrease over time. After an extraction of 12 h, the concentration of molecules decreased toward the resolution limit (Fig. 1C). Especially, for Zendura material that decrease occurred already after a period of 1 h. The summary of all single component-related concentrations can be found in the supplementary material (supplementary Tables S2 and S3).

### Isothermal microcalorimetry determination of the antimicrobial effect

**Figure 2:**
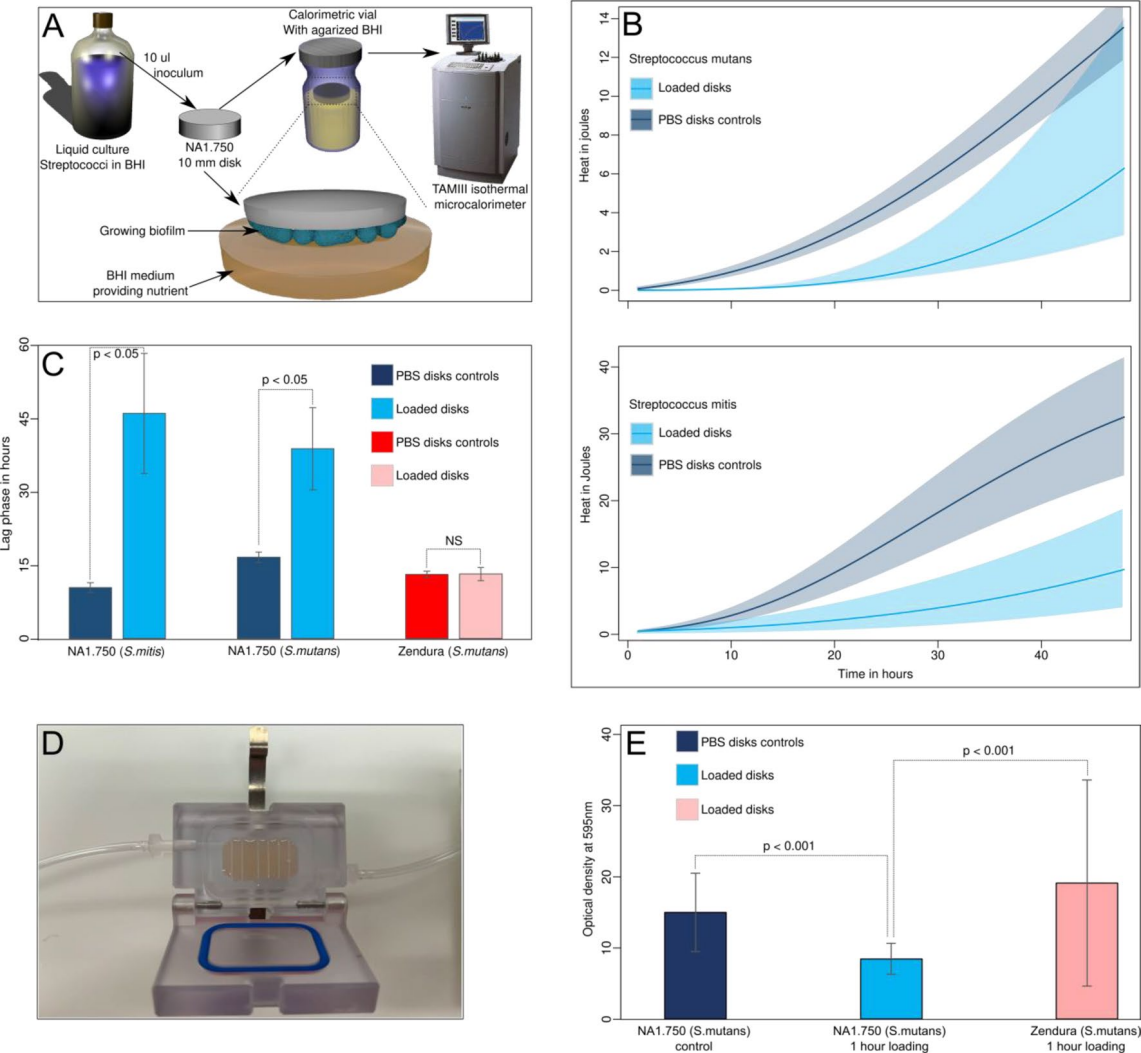
(**A**) Simplified sketch showing the experimental procedure to assess the antimicrobial activity of loaded thermoformable materials (NA1.750 (blue) and Zendura (red) as control) using isothermal microcalorimetry to measure biofilm growth and metabolic heat (see details in the main text) under static conditions. (**B**) Growth of *S. mutans* (top) and *S. mitis* (bottom) biofilms on NA1.750 material loaded with the care solution (both light blue) containing EOs or with PBS as control. Note the delay in growth in both cases. (**C**) Lag phase duration (i.e., delay in growth) of biofilms calculated using the Gompertz growth model and expressed in hours for the different materials loaded with EOs or PBS as control. (**D**) Flow cell setup used to assess the development under constant liquid flow. (**E**) Amount of biofilm formed on thermofoil material disks in the flow cell (see **D**) in 16 h quantified using the crystal violet assay. In C, and E the vertical bars represent the standard deviation, differences between groups are indicated by the *p* value; NS indicates no significant differences.

The selected bioactive molecules solved in a care solution are loaded into the different thermoformable polymer materials (NA1.750 and Zendura) for a period of 1 h. The antimicrobial effect of the loaded materials was assessed using a solid medium in combination with isothermal microcalorimetry and compared to unloaded control materials placed in PBS for the same time (Fig. 2A).

Utilizing loaded NA1.750 material, the growth of both *S. mutans* and *S. mitis* was distinctly delayed (Fig. 2B). Such delay has been clearly quantified using growth models in Table 1, where the lag phase (i.e., the time until exponential growth) showed an increase of approximately 22 h for *S. mutans* and 35 h for *S. mitis* (Fig. 2B). After this initial growth delay, the observed growth rates remain similar to unloaded NA1.750 (Table 1). On the contrary, a loading of reference material Zendura with the care solution had no effect on the biofilm formation, as no delay of the bacteria growth was observed compared to unloaded PBS samples (Fig. 2C). This correlates with the findings from the GCMS measurements, demonstrating that Zendura showed only minor absorption capabilities for the bioactive molecules relevant to bacteria growth delay (Fig. 1).

**Table 1:** Growth parameters calculated fitting the Gompertz model on the microcalorimetry data.

Sample	Growth rate (h^-1^)	Lag phase (h)	Q (J)	n
NA1.750 loaded (*S. mutans*)	0.54 ± 0.24	38.57 ± 8.35^a^	48.71 ± 5.13	3
NA1.750 unloaded (*S. mutans*)	0.44 ± 0.04	16.52 ± 1.06	36.54 ± 3.30	3
NA1.750 loaded (*S. mitis*)	0.80 ± 0.22	45.75 ± 12.20^a^	133.57 ± 35.04^b^	3
NA1.750 unloaded (*S. mitis*)	0.93 ± 0.24	10.36 ± 1.00	45.55 ± 11.37	3
Zendura loaded (*S. mutans*)	0.58 ± 0.28	13.11 ± 1.35	19.10 ± 9.19	3
Zendura unloaded (*S. mutans*)	0.47 ± 0.04	13.05 ± 0.65	15.31 ± 1.31	3

Values are expressed as mean ± standard deviation.

^a^Indicate a significant difference (*p* < 0.05) between the treated and non-treated groups.

^b^A thick biofilm formed at a later stage in one sample increasing heat production. The calculations of lag phase and the growth rate were not affected so the sample was kept in the analysis.

### Flow chamber evaluation of *S. mutans* biofilm formation

The caries-related biofilm formation with *S. mutans* using conditions mimicking the sheer stress comparable to saliva flow in the oral cavity was assessed by flow chamber experiments (Fig. 2D,E). The loaded NA1.750 material showed the lowest biofilm formation on its surface. Unloaded NA1.750 material was more prone to biofilm colonization showing an increase of 78% compared to its loaded counterpart (i.e., 100% reference) (*p* < 0.001, n = 96). This emphasized that the treatment with care solution containing bioactive molecules was effectively adding antimicrobial properties to the NA1.750 material. Loaded Zendura material exhibited the highest amount of surface biofilm formation after 16 h with an increase of 127% compared to loaded NA1.750 material (*p* < 0.001, n = 96). Again, this result correlates with the GCMS data and the related low absorption capabilities of bioactive molecules found for the Zendura material. Microscopic observations on loaded and unloaded NA1.750 also confirmed the reduction of biofilm formation for *S. mutans* and *S. mitis* (see supplementary Figs. S4 and S5).

### Cytotoxicity

**Figure 3:**
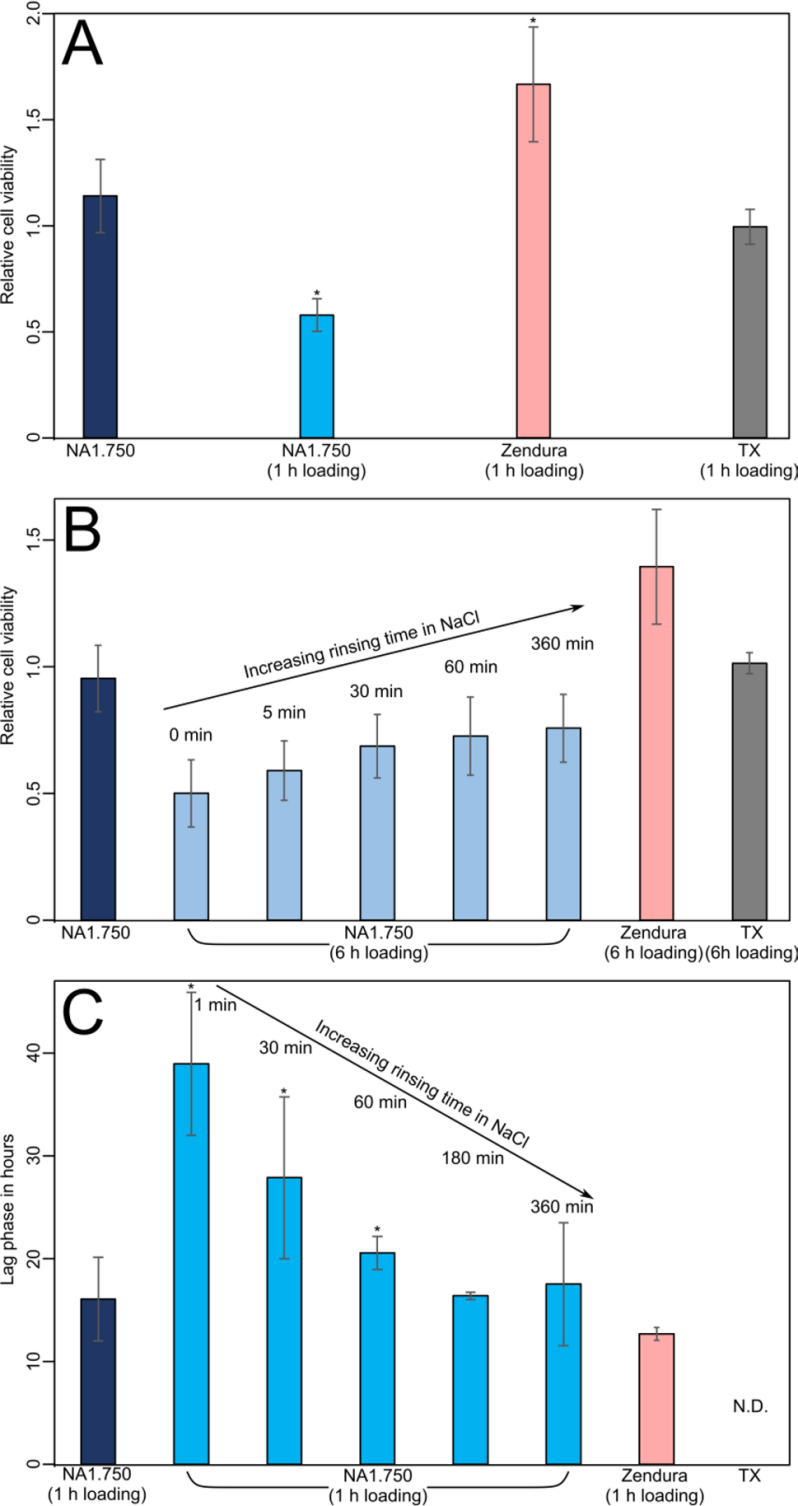
(**A**) relative cell viability and cytotoxicity of the thermofoil materials (measured according to the ISO 10993-5 standard). Thermanox (TX) was used as a control and values have been normalized to TX. (**B**) Evolution of cytotoxicity of the loaded NA1.750 material with increasing rinsing time. Thermanox (TX) was used as a control and values have been normalized to TX. (**C**) Evolution of antimicrobial efficacy of the loaded NA1.750 material with increasing rinsing time. Zendura is added for comparison purposes. In (**A**, **B**, and **C)** the vertical bars represent the standard deviation. * indicate significant differences with unloaded material and TX.

Cytotoxicity tests were performed to ensure the biological safety of such loaded polymer material. For this, the thermoformable polymers described above were loaded for 1 h, and briefly rinsed and their cytotoxic response was investigated towards human gingival fibroblast (HGF-1) cells according to the ISO 10993-5 (see details in materials and methods section). Thermanox (TX—polystyrene) was used as a baseline material known for its good biocompatibility. Unloaded thermoformable materials showed high cell viability. The NA1.750 material had a relative cell viability of 115% compared to the Zendura material with a relative cell viability of 168% (Fig. 3A). When performing the assay with loaded NA1.750 material, the bioactive molecules extracted from the material decreased the viability of HGF-1 for an indirect exposure of 24 h to 58%.

### Rinsing experiments

Simulating the saliva flushing and the related extraction of antimicrobials in the oral cavity, the NA1.750 loaded material was rinsed with water, for increasing time before the cytotoxicity assessment. With increasing rinsing time, the cytotoxicity decreased (Fig. 3B). Compared to the Thermanox baseline, unrinsed NA1.750 samples showed a viability of 49 ± 13%, as samples rinsed for 5 min already showed a viability of 58 ± 12% (*p* = 0.005, n = 54). After 30 min and 60 min, the viability increased to 68 ± 12% (*p* < 0.001, n = 54) and 72 ± 15% (*p* < 0.001, n = 54), respectively. This relative cell viability did not increase substantially after 1 h and remained at 75 ± 13% (*p* = 0.26, n = 54).

As rinsing has been shown to release the bioactive molecules from the NA1.750 loaded material, reduced antimicrobial activity was expected. Therefore, similar rinsing experiments were performed to assess the remaining antibacterial efficacy against *S. mutans* after rinsing the loaded NA1.750 polymer. A significantly longer lag phase 39 ± 7 h is still observed after 1 min rinsing (*p* = 0.007, n = 6). With longer rinsing time, the lag phase decreased to the baseline value as the rinsing time increased (Fig. 3C). After 3 h of rinsing, no differences in lag phase was observed compared to unloaded materials (Fig. 3C) (*p* = 0.45 and *p* = 0.37 for 3 and 6 h respectively). As for the previous measurement, the growth rate did not show significant changes (*p* = 0.61, n = 18).

### Mechanical characterization

**Figure 4:**
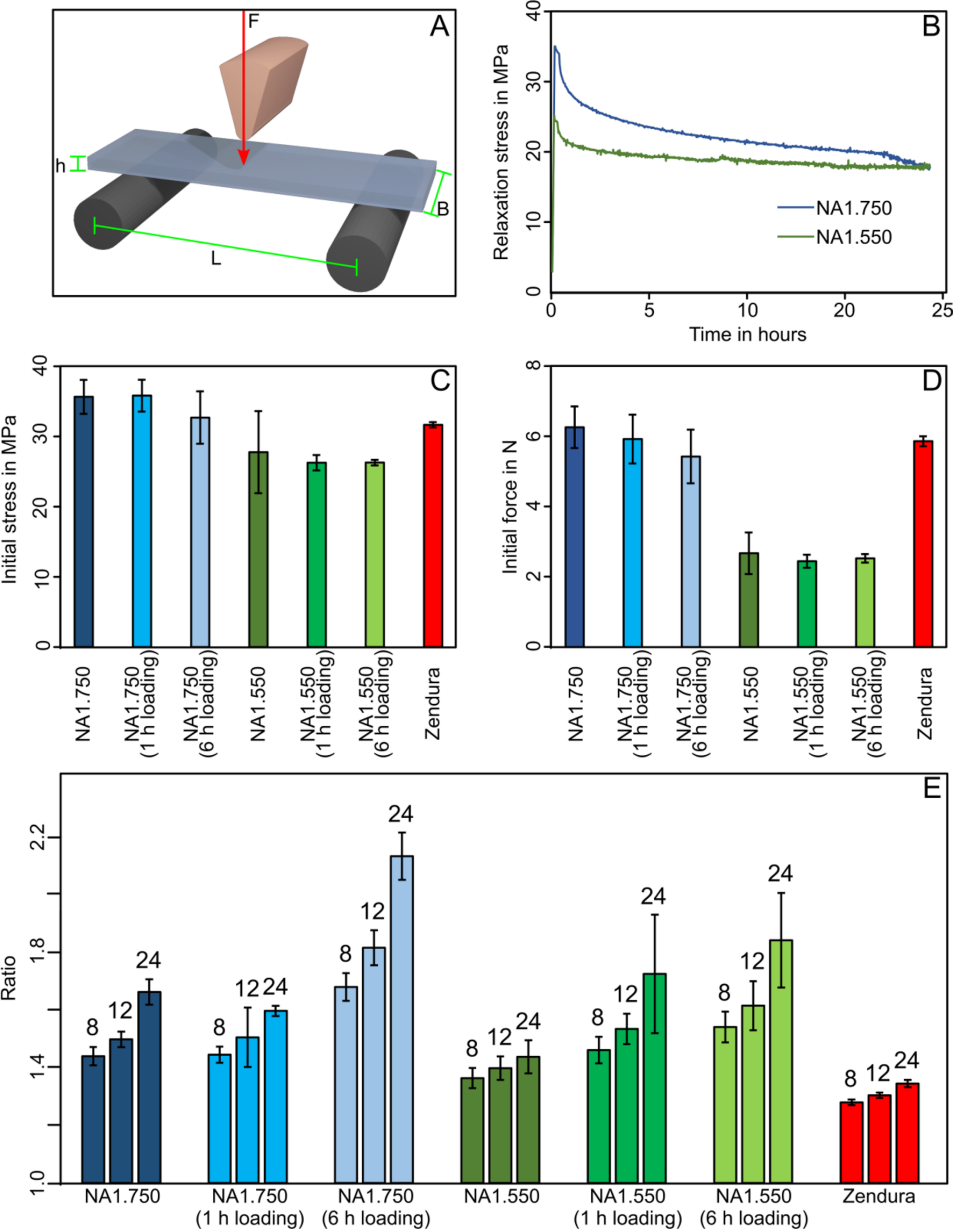
(**A**) Scheme of a 3-point bending setup with parameters described in Eq. (2–3). (**B**) Example stress relaxation measurement of thermoplastic materials during static bending conditions applied for 24 h. (**C**) Measured initial (maximum) stresses and, (**D**) Measured initial (maximum) forces of bended thermofoil material after 24 h static bending under wet conditions. (**E**) This Figure shows the stress ratio of the tested samples after 8 h, 12 h, and 24 h. The ratio was calculated by dividing the initial stress and the stress after 8 h, 12 h, and 24 h bending. In (**C**, **D**, and **E**) the vertical bars represent the standard deviation.

The 3-point bending tests (see Fig. 4A) characterize the mechanical properties and time-dependent stability of the thermoformed and loaded polymers (Zendura; as well as loaded and unloaded NA1.550, NA1.750) based on stress relaxation detection over time (see example in Fig. 4B). The mean maximum forces (initial force) and the mean maximum stresses (initial stress) with their corresponding standard deviation are represented in Fig. 4C,D. Although small variations were observed, the loading time did not significantly influence the initial force (*p* = 0.916, n = 25) nor the initial stress (*p* = 0.465, n = 26). NA1.750 and NA1.750 material loaded for 1 h with bioactive molecules show similar initial stresses with 35.35 ± 2.38 MPa and 36.28 ± 2.29 MPa and similar initial forces with 6.1 ± 0.8 N and 5.9 ± 0.7 N, respectively. A 6 h loading of the NA1.750 6 h resulted in a lowered initial stress of 33.12 ± 3.77 MPa and initial force of 5.4 ± 0.8 N comparable to that of Zendura showing an initial stress of 32.06 ± 0.38 MPa and a force of 5.8 ± 0.1 N. The significant lower initial force and stress values of the NA1.550 material, loaded and unloaded, are due to the lower material thickness compared to NA1.750 and Zendura (*p* < 0.001, n = 25, and *p* < 0.001, n = 26 respectively). The stress ratios of the tested samples are shown in Fig. 4E. The ratio was calculated by dividing the initial stress and the stress after 8 h, 12 h, and 24 h bending. Zendura presented the lowest ratio values and, therefore, the lowest stress relaxation of 1.28 ± 0.02 and 1.35 ± 0.02 after a time period of 8 h and 24 h. NA1.750 exhibited stress ratios of 1.44 ± 0.32 to 1.66 ± 0.44, for 8 h to 24 h of stress relaxation. Finally, the loading with bioactive molecules had no significant impact on the mechanical stability of NA1.750 material when loaded for 1 h showing a slightly lower stress relaxation after 24 h of 1.60 ± 0.03. For a 6 h loading time, the overall stress relaxation ratios of NA1.750 increased to 2.14 ± 0.08 after 24 h compared to the unloaded material system. This pronounced loss in mechanical stability at a loading time of 6 h does not apply to NA1.550.

Thus, the loading with bioactive molecules through soaking in care solution does not lead to a significant increase in stress ratios when loaded for 1 h and therefore, seems to have no distinct negative impact on the mechanical stability of the NA1.750 and NA1.550 materials with its intended use as clear aligners.

## Discussion

Absorption and release of EOs by the cellulose-based thermoformable material resulted in an antimicrobial effect against *S. mutans* and *S. mitis* achieved by loading the material with a combination of 5 bioactive compounds extracted from EOs. In addition, the material kept its mechanical properties and remained safe toward human gingival fibroblasts. The antimicrobial effect cannot be observed in gold-standard PETg or TPU-based polymers (e.g. Zendura) as bioactive agents cannot be loaded with essential oils (only a minute fraction might adhere to their surface).

The detected antimicrobial effect of this study compares well to promising in vivo studies obtained with single bioactive molecules or EOs from which those molecules are purified. For example, plaque and gingival index reduction obtained with cinnamon EO has been shown to be comparable to the reduction obtained with chlorhexidine gluconate (0.2%)^22^. Indeed, the main component of cinnamon EO representing from 51 to 84% is cinnamaldehyde^26,27^, also present in the solution studied here. Cinnamaldehyde has also been shown to inhibit the acidification of *S. mutans* biofilms, which leads to less favorable conditions for the bacteria and thus, to reduced biofilm formation; in the combination of the effect which cinnamaldehyde has on the cell surface of *S. mutans* by increasing its hydrophobicity leading to reduced ability to co-aggregate, this EO plays an important role in the overall inhibition and reduction of cariogenic biofilm formation^28^.

Other EOs such as eucalyptol (1,8-cineole) and limonene often associated with cinnamaldehyde in natural extracts^19,26,27^, are also part of the composition of the solution used for loading the NA1.750 material. Eucalyptol (1,8 cineole) has demonstrated antimicrobial as well as antibiofilm properties against *S. mutans*^29^; while limonene has been shown to possibly inhibit acid resistance of *S. mutans* and biofilm formation at sub-MICs level without necessarily affecting the growth of the bacteria^30^. Furthermore, similar studies show that such bioactive molecules or EOs can have a broader impact on oral health by inhibiting the growth of pathogens responsible for gingivitis and periodontitis, such as *Porphyromonas gingivalis*^19^ or by limiting the inflammation caused by *Aggregatibacter actinomycetemcomitans*^31^.

In the presence of 5 natural antimicrobials, the cellulose-based thermoformable material inhibited the growth of streptococcal biofilm on the surface, delaying the growth onset by a period of 22 h compared to unloaded material under static conditions. In addition, the biofilm formation during such time intervals is strongly decreased as well (at t = 16 h) under sheer stress comparable to saliva-flow conditions. Such inhibition periods are longer compared to the typical wearing periods of invisible braces between two cleaning or potential loading events (approximately 8 h). Therefore, when used and loaded at appropriate intervals, less biofilm formation can be expected on dental brace manufactured from such material, that in turn will also lead to reduced risk of plaque formation and caries.

Concerning the use in the context of oral care, the cytotoxicity of the material was deemed acceptable according to European guidelines. Indeed, the test method used a 24 h extraction in a relatively small volume (0.5 mL), which would represent a worst-case scenario. However, in the oral cavity, the aligner is subjected to a constant flow of saliva (0.5 to 1.5 L day^−1^)^32^. Thus, assuming that extraction volumes would be matched, the concentrations of EOs would, in turn, be reduced by 1000 times (using minimal saliva production). The slow-release kinetics of bioactive molecules allow the application to operate at low concentrations, orders of magnitudes away from the food intake limits of the single compounds set by the European chemical agency (https://echa.europa.eu/); therefore, not negatively affecting the surrounding tissues. Concerning the potential “cocktail” effect of the different bioactive molecules present in the loading solution and, finally, in the impregnated material, it must be noted that many of these compounds are often found together in EOs (for example in cinnamon EO and wintergreen EO^19,26,27,33^). The fact that some EO extracts have been shown to have a more antimicrobial effect than their isolated components suggests that there are at least some additive effects and/or synergies between those components that should be further investigated^8,34^. The presented results are promising and should be reproduced in prospective in vivo studies. Indeed, the concentrations in loaded invisible brace materials are in line with studies on murine periodontitis models showing that oral intake of cinnamaldehyde at a concentration of 8 to 16 mg kg^−1^ day^−1^ significantly decreases the microbiota dysbiosis and host inflammatory response while promoting osteogenic induction effects. Furthermore, the same study emphasizes that the oral intake of cinnamaldehyde remained safe for the animals^35^. Potentially, that qualifies invisible braces loaded with bioactive molecules as anti-inflammatory dental appliances for preventive oral care.

Concerning the mechanical properties of the invisible braces, it must be noted that the loading of both cellulose-based thermoformable materials (NA1.750 and NA1.550) did not result in a substantial decrease in mechanical stability. Hereby, a constant deflection applied to the polymers simulates the interaction between the invisible brace and teeth over time. The detected initial forces of the Naturaligner material system ranging from 2 to 7 N are comparable to the in-vitro results of gold standard thermoplastic materials applied in clear aligner therapies^36,37^. In addition, both cellulose-based materials loaded with antimicrobials can provide comparable initial forces to initially move teeth within clear aligner therapies equivalent to gold-standard materials such as Zendura. The mechanical stress stability (stress resistance) qualifies how forces are translated to the teeth over time during a clear aligner therapy^38^. The stress resistance of the loaded Naturaligner materials is comparable to relaxation behaviors of gold-standard materials like Duran, Zendura, and other similar materials^39^, and thus deliver sufficient forces over time to convert e.g. lateral and distalization movements during clear aligner therapies^40^.

Finally, we must emphasize the strengths and weaknesses of the study. Or study provides a rather complete set of experiments delivering a bigger picture of the potential for invisible braces with the antimicrobial coating but also potentially as a device for drug delivery in the oral cavity. Still, the limitations of our study lie in the number of strains investigated. Indeed, the oral cavity is inhabited by more than 700 species^41,42^ and our models only includes two of those belonging to the same genus. Further, studies should include different microbes to extend our observations. Interesting candidate could belong to *Actinomyces oris* and related species being early colonizers responsible for gingivitis and recently recognized as an important player in periodontitis^43–45^. It must be noted that the current setup does not fully allow working with anaerobic conditions and higher CO_2_ concentrations that are commonly encountered in the oral cavity. This currently limits this study to strains that tolerate oxygen well and do not require higher pCO_2_. However, further investigation on periodontal pathogens will require such conditions. In addition, in some cases technical limitations (i.e., number of measuring channels) impacted the number of samples investigated. The limited number of replicates is clearly a limitation of this study. To further investigate such limitation, a retrospective power analysis was performed. It showed that with the large effect observed the statistical analysis were sufficiently powered (power > 0.82 for calorimetry and power > 0.99 for all other analyses). Although our results are promising, they mostly advocate for more research and in particular for further animal studies or clinical trials with additional in-vitro investigations on wider range of microbes isolated from the oral cavity across a wider range of conditions. In particular, pathogens responsible for gingival inflammation and periodontitis should be given more attention. Finally, saliva-extracted inflammation markers such as MMP-8/9 could also be valuable.

## Conclusion

In contrast to high-concentrated mouthwash or other liquid disinfectants like chlorohexidine, the continues and targeted releases of antimicrobial agents from cellulose-based dental braces towards the teeth and soft tissue allows the use of body-friendly and non-cytotoxic doses still achieving significant antibiofilm efficacy. Currently, for thermoformable dental brace material, such antimicrobial effect with the presented natural substances is only realized through the combination with cellulose-based Naturaligner material. The loaded materials showed stable mechanical characteristics allowing them to serve their traditional intended use as clear aligners, nightguard, or retainer. In this context, the integration of antimicrobial essential oils in customized invisible dental braces illustrates an efficient approach towards preventive dental care through orthodontics treatments. Overall, in addition to its antimicrobial effects, the loaded cellulose-based thermoformable material presented here provides a valuable platform for drug delivery in a personalized manner directly to the tooth surface and gingiva.

## Methods

### Materials

The cellulose-based multilayered thermoformable material (Naturaligner; Bottmedical; Switzerland), having a thickness of 750 μm (NA1.750) and 550 μm (NA1.550) was used. These thermoformed foils (40 s and 35 s heated at 220 °C in Biostar; Scheu Dental; Germany) were cut into discs with a diameter of 10 mm to be used in the subsequent antimicrobial testing experiments. Zendura FLX (Bay Materials; Fremont; CA; U.S.A.) was thermoformed at 50 s, cut into discs with a diameter of 10 mm, and tested as reference material. The thermoformed materials were impregnated with bioactive molecules of essential oils by placing disks (no more than five disks) in 10 mL of a solution containing the mixture of four bioactive molecules for one h with gentle shaking (15 rotations per minute on a roller shaker). The water-based solution containing essential oils and bioactive molecules contained cinnamaldehyde, methyl-salicylate, eucalyptol, limonene, and trans-anethole with a ratio of 1/20 to polyglycerol-4 laurate/sebacate solubilizer in water (care solution). A brief rinsing (ca 5 s) in PBS followed by drying on sterilized paper tissue was applied to remove unbound essential oils. All chemicals were obtained from Sigma Aldrich (Buch; Switzerland).

### Gas chromatography-mass spectrometer

The absorption and release of the five bioactive molecules of the care solution by the NA1.750 thermoformable material were measured using GCMS. An Agilent GCMS System (Santa Clara; CA; U.S.A.) using an HP-5MS UI capillary column (30 m × 0.250 mm, 0.25 µm thickness, Agilent Technologies; Santa Clara; CA; U.S.A.). The initial oven temperature was 75 °C for 2.25 min, and the temperature was increased to 300 °C at a rate of 15 °C min^−1^ and held at this temperature for 3 min. Helium was used as carrier gas at a flow rate of 1.0 mL min^−1^. The injection volume was 1 µL at a 20:1 split ratio. The injector temperature was 300 °C, the transfer line temperature was 320 °C, and the MS Source temperature was 240 °C. Electron impact ionization (EI) was carried out at 70 eV. Detection was operated under the selected ion monitoring (SIM) mode. The molecular ion peak was used for quantitation except for Triacetin (145.1 m/z) and Carvacrol (135.1 m/z). The Instrument was operated with the MassHunter GC/MS Acquisition Software (B07.06.2704). Processing, interpreting, and quantifying the measured data were performed using MassHunter Quantitative Analysis (Agilent Technologies; Santa Clara; CA; U.S.A.—Version B.09.00/Build 9.0.647.0).

Calibration was performed using trans-cinnamaldehyde (≥ 95%), (+)-carvone (≥ 98%), trans-anethole (99%), (R)-(+)-limonene (97%), eucalyptol (99%), methyl salicylate (≥ 99%), carvacrol (99%) and triacetin (99%) obtained from Sigma Aldrich (Buch; Switzerland) and HPLC-Grade ethanol supplied by Fisher Scientific (Reinach; Switzerland). The stock solution contained a mixture of all standard samples at an individual concentration ranging from 588 µg mL^−1^ (limonene) to 826 µg mL^−1^ (methyl salicylate) in ethanol. Further dilution with ethanol gave the working solutions that were used to evaluate the detector response linearity and assign a calibration curve to each of the compounds of interest. Calibration curves: D-limonene: 4 points (0.37–18 µg mL^−1^, R^2^ = 0.9987); eucalyptol: 4 points (0.41–20 µg mL^−1^, R^2^ = 0.9998); methyl salicylate: 5 points: (0.52–50 µg mL^−1^, R^2^ = 0.986); carvone: 4 points (0.40–19 µg mL^−1^, R^2^ = 0.9975); cinnamaldehyde: 5 points (0.46–44 µg mL^−1^, R^2^ = 0.9778); *trans*-anethole: 4 points (0.43–21 µg mL^−1^, R^2^ = 0.9910), carvacrol: 5 points (6.9–172 µg mL^−1^, R^2^ = 0.9972); triacetin: 4 points (0.51–24 µg mL^−1^, R^2^ = 0.9926). The determined accuracy typically was between 80 and 120% for concentrations above 5 µg mL^−1^ and significantly decreased for concentrations below 1 µg mL^−1^.

### Extraction of substances from the loaded polymer samples

After loading 3 cm^2^ of the thermoformed polymer samples in 5 mL of care solution, the samples were briefly rinsed in water, and their bioactive molecules content was extracted in 5 mL of 40% ethanol for up to 24 h. Bioactive molecules were extracted on a shaker (Type 44/44, Exakt) with 100 rotations·min^−1^. Due to the high number of samples needed for the time series (loading and release), only 1 batch of samples was analyzed. However, we assume that the larger surface used compensates for possible variations. Furthermore, the accuracy of the measurement is described above and is acceptable for such type of investigation.

### Isothermal microcalorimetry determination of the antimicrobial effect

For the isothermal calorimetry experiments and the determination of the antimicrobial activity of loaded polymer material, *Streptococcus mutans* (ATCC 25175) and *Streptococcus mitis* (ATCC 49456) were used as oral microbiome representatives (LGCpromochem; Wesel; Germany). These two streptococci are considered potential early colonizers for invisible braces used for up to 12 h (i.e. roughly overnight). Before use, overnight cultures of streptococci were made in brain–heart infusion (BHI—Sigma Aldrich; Buchs; Switzerland) using a frozen aliquot as inoculum. A fraction of the aliquot was plated on BHI agar to ensure the purity of the strain present in the aliquot. When required, the density of the culture was measured by appropriate plating dilution on agarized BHI (aBHI—agar and BHI from Sigma Aldrich; Buchs; Switzerland).

For the calorimetric measurement, 10 µL of the preculture was placed on a loaded thermoformable material disc. The inoculated side of the discs was placed facing solid BHI prepared in 20 mL calorimetric vials. Slight pressure was applied on the backside of the disc until the liquid was evenly distributed through the surface between the disc and the agar. The vials were sealed and introduced in a calorimeter (TAM III and TAM air; Waters/TA instruments; Delaware; USA). After submitting the vials in the calorimeter and a period of 1 h for thermal equilibration, the heat flow was recorded until the signal returned to baseline or for at least 96 h. Negative controls were made with discs that were exposed to PBS for 1 h. Similarly, sterility during the measurements was checked by using an unloaded and uninoculated disc that went through the same procedure. All calorimetric measurements were performed in triplicates using sterile reagents and material with aseptic techniques.

At the end of the experiment, data were collected and transformed into an ASCII file using TAM assistant (Waters/TA instruments; Delaware; USA). The raw heat flow curves were integrated to obtain total heat over time curves similar to a growth curve. These data were then fitted using the modified Gompertz model as rearranged by Zwietering et al.^46^ (Eq. 1) and limited to use the following parameters: μ_max_ the maximum growth rate, λ the duration of the lag phase, Q_t_ the heat produced at time t, and Q_max_ the maximum heat. All calculations were performed using R and the grofit package^47,48^.

1 $$Q_{t} = Q_{max} \cdot e^{-e\left[\left({\frac{\mu_{max} \cdot e}{{Q_{max} }}}\right)\cdot(\lambda-t)+ 1\right]}$$

### Flow chamber evaluation of *S. mutans* biofilm formation

To confirm calorimetric results in a system with the liquid flow (to mimic sheer stress comparable to saliva flow in the oral cavity), flow chambers (Minucells; Bad Abbach; Germany) were used (Fig. 2D).

One colony of *Streptococcus mutans* (ATCC 25175) grown on Columbia blood agar plate (Columbia agar; BBL; Becton Dickinson; Basel; Switzerland) was inoculated into 25 mL of Todd Hewitt medium (BBL; Becton Dickinson; Basel; Switzerland) supplemented with 0.5% sucrose (Sigma Aldrich; Buchs; Switzerland) and incubated aerobically at 37 °C for 22 h. *S. mutans cells* were harvested in the stationary growth phase, washed with physiological saline (Sigma Aldrich, Buchs, Switzerland), and resuspended in simulated body fluid (SBF) (7.996 g NaCl, 0.35 g NaHCO_3_, 0.224 g KCl, 0.228 g K_2_HPO_4_·3H_2_O, 0.305 g MgCl_2_·6H_2_O, 0.278 g CaCl_2_, 0.071 g Na_2_SO_4_, 6.057 g (CH_2_OH)_3_CNH_2_ dissolved in 1 L of ultra-pure water, pH adjusted to 7.25 with 1 mol L^−1^ HCl, all chemicals from Sigma Aldrich (Buchs; Switzerland)^49^. Discs of thermoformable material (NA1.750) previously loaded with the care solution as well as the same disc without care solution loading and Zendura FLX (Bay Materials; Fremont; CA; U.S.A.) were coated with pooled saliva (anonymously collected saliva was pooled and filtered through 70 µm filters, centrifuged at 22,000 g for 40 min at 4 °C; the supernatant was collected and filter-sterilized by applying two consecutive filters 0.45 and 0.22 µm) prior to each experiment for 15 min at room temperature. Circulating bacteria (approximately 2·10^8^ CFU mL^−1^) were allowed to adhere to the protein-coated materials at 37 °C for 16 h in an aerobic flow chamber system containing a bacterial reservoir and flow chamber harboring one group of specimens placed on a shaker (180 rpm to keep the solution homogeneous), peristaltic pump running at 0.8 mL min^−1^ to mimic sheer stress comparable to saliva flow in the oral cavity and a waste collection bottle for used bacterial suspension. Each flow chamber had space for 24 specimens, and all groups were run twice, resulting in n = 48 for all of each three sample groups.

After that, the discs were carefully removed from the flow chamber, dipped gently in physiological saline, and air-dried in 24-well plates. The dried biofilms were stained with 0.5% crystal violet solution for 10 min in the dark and after that, the excess dye was removed by dipping the specimens in 3 L of ultrapure water to remove any unbound dye. After air drying, the specimens were destained by ethanol absolute, and the absorption was measured at 595 nm with a spectrophotometer (BioTek Synergy 2, BioTek Instruments AG, Luzern, Switzerland), ethanol absolute was used as a blank measurement. The experiments were performed with sterile reagents and material using aseptic techniques.

### Cytotoxicity assays

To ensure that the loaded polymer materials do not have a cytotoxic effect on cells found in the gingiva, the cell viability of human gingival fibroblast (HGF-1) was evaluated. HGF-1 (ATTC American Type Culture Collection (LGCpromochem; Wesel; Germany)) was cultivated in cell culture medium in an incubator (CB 220; Binder) at 37 °C and 5% CO_2_. The medium consisted of Dulbecco’s Modified Eagle Medium (DMEM high glucose; Sigma-Aldrich; Buchs; Switzerland) with 1 mL penicillin–streptomycin (Sigma-Aldrich; Buchs; Switzerland), 1 mL sodium-pyruvate (Gibco; Thermo Fisher Scientific; Reinach; Switzerland), 1 mL L-glutamine (Gibco), 1% amphotericin B solution (Sigma-Aldrich; Buchs; Switzerland), and 10 mL fetal calf serum (FCS superior; Bioswisstech; Schaffhausen; Switzerland) added per 100 mL. Passages 4 to 9 of human gingival fibroblast cells (HFG-1) were used for the experiments. Before reaching complete confluency, cells were rinsed with phosphate-buffered saline (PBS—Sigma-Aldrich; Buchs; Switzerland) two times, then 1.5 mL 5% trypsin/2% ethylene diamine tetraacetic acid (EDTA) solution was added to detach the cells. Experiments were conducted using an indirect approach according to ISO standard 10,993–5 (annex C,D). Polystyrene discs (Thermanox Plastic Coverclips; Nalgene Nunc International; Rochester; NY; USA) served as negative and 90% ethanol as a positive control. Thermoformable material disks with a diameter of 12 mm were prepared and loaded as described above and stored under sterile conditions in 24-well plates until use.

Thermoformable material (NA1.750) discs were incubated at 37 °C in 500 µL of full medium for 24 h in 24-well plates. Meanwhile, 10^4^ cells were seeded per well to 24-well plates and incubated for 24 h. After 24 h, the cell culture medium was removed, cells were washed twice with PBS, the medium of the incubated samples was added, and cells were incubated for another 24 h. A total of 9 specimens were tested per group for both experimental set-ups.

Afterward, a WST-1 cell viability assay (Cell Proliferation Reagent WST-1; F. Hoffmann-La Roche; Basel; Switzerland) was conducted. Cell medium was therefore aspirated, and 500 µL of WST-1 solution mixed with cell medium (1:10) was added to each well. The specimens were then incubated for another 2 h. After incubation, 3 × 100 µL of WST-1 solution per well was transferred into a 96-well plate, and the optical density was measured (RT-2100C Microplate Reader; Rayto Life and Analytical Sciences; Shenzhen; China) at a wavelength of 420 nm. The results were normalized using the results of Thermanox (TX—polystyrene) as a reference material that is known for its good biocompatibility to determine the relative cell viability. The experiments were performed with sterile reagents and material using aseptic techniques.

### Rinsing experiments

As rinsing the loaded thermoformable material (NA1.750) releases bioactive compounds from the cellulose-based layer, the effect of rinsing was evaluated on the cytotoxicity as well as on the antimicrobial efficacy using the same methodologies as described above (i.e., isothermal microcalorimetry and WST-1 assay). The loading of the thermoformable material was performed by putting discs of the thermoformable material in the care solution (5 discs for 10 mL of solution) and incubating those discs under slow agitation (15 rotations per minute) for 1 h. Rinsing was performed in the same condition using PBS (for isothermal microcalorimetry) or 0.9% NaCl solution (for cytotoxicity determination). Different rinsing times were chosen in a range from 1 min to 6 h. A total of 9 specimens were tested per rinsing time. The experiments were performed with sterile reagents and material using aseptic techniques.

### Mechanical characterization

To ensure that the essential oils bioactive molecules loading was not negatively affecting the mechanical properties of the thermoformable material, mechanical tests were performed as follows. Relaxation tests were performed on an electromechanical universal testing machine (FMT-313 Alluris; Freiburg im Breisgau; Deutschland) equipped with a load sensor (50 N) recording time-resolved force loading. The specimens were first laser-cut from thermoformed NA1.750, NA1.550, and Zendura (Bay Materials; Fremont; CA; U.S.A.) materials using a laser cutting procedure (40W cw CO_2_ laser; Glowforge; Seattle; WA; U.S.A.) in four samples with a size of 40 × 15 mm each.

The 3-point bending test was performed to measure their static properties, e.g. maximum force (initial force) and maximum stress (initial stress) and their stress-relaxation over a 24 h loading period^50^. The span was set to 22 mm. Due to the varying material thickness of samples after thermoforming the vertical deflection of the bending tip was set to 2 mm to achieve an internal elongation of 1.6%^49^. The thickness of each sample was measured after thermoforming with a micrometer screw at three different locations and the mean value was calculated. The force (N) was recorded every 59 s for 24 h.

The relaxation stress is calculated from Eq. (2–3). The stress ratio between initial stress and the reduced stress after periods of 8, 12 and 24 h of relaxation is determined.

2 $$\sigma_{eng} = \frac{3 \cdot F \cdot L}{{2 \cdot B \cdot h^{2} }}$$

and

3 $$\epsilon_{eng} = \frac{6 \cdot h \cdot \Delta L}{{2 \cdot B \cdot h^{2} }}$$

σ is the engineering stress (MPa) and ε is the engineering strain. In those equations, L is the support span length = 22 mm, F is the force (N), B is specimen width (15 mm), and h specimen thickness (mm).

Thermoformed polymer samples were placed either in water (Zendura—Bay Materials; Fremont; CA; U.S.A.) or in the antibacterial care solution to evaluate the influence of loaded antimicrobial substances with respect to the material’s mechanical stability. All mechanical testing assessments were performed at least in triplicate.

### Statistical analysis

For descriptive analysis and comparison between parameters calculated in the previous sections, data were collected in spreadsheet software. The normality of the results was tested using the Shapiro–Wilk test for a small sample size. The Student’s t-test or the Wilcoxon test was applied where appropriate to assess statistically significant differences between the loaded and unloaded groups of materials. After distributional assumptions (normality and comparable variances) were checked, a two-way ANOVA was used to assess the importance of thickness and loading time on the mechanical parameters of the material. The level of significance was set to *p* < 0.05. All data were processed using R (version 3.6.3)^47^. All results showed in the text are expressed as mean ± standard deviation.

### Supplementary Information

Supplementary Information.

## Data Availability

Data can be obtained from the authors directly or by contacting the corresponding author.

## References

[1] Brody H (2021). Science opens wide for oral health. Nature.

[2] Wu H (2022). The periodontal pathogen fusobacterium nucleatum exacerbates Alzheimer’s pathogenesis via specific pathways. Front. Aging Neurosci..

[3] Plackett B (2021). Research round-up: Oral health. Nature.

[4] World Health Organization. WHO | Oral health. *Who* (2016).

[5] Mummolo S (2020). Salivary concentrations of Streptococcus mutans and Lactobacilli during an orthodontic treatment. An observational study comparing fixed and removable orthodontic appliances. Clin. Exp. Dent. Res..

[6] Prabuseenivasan S, Jayakumar M, Ignacimuthu S (2006). In vitro antibacterial activity of some plant essential oils. BMC Complement. Altern. Med..

[7] Gende LB, Floris I, Fritz R, Martin Javier Aras EGU (2008). Antimicrobial activity of cinnamon (*Cinnamomum zeylanicum*) essential oil and its main components against *Paenibacillus larvae* from argentine. Bull. Insectol..

[8] Chouhan S, Sharma K, Guleria S (2017). Antimicrobial activity of some essential oils—Present status and future perspectives. Medicines.

[9] Becerril R, Gómez-Lus R, Goñi P, López P, Nerín C (2007). Combination of analytical and microbiological techniques to study the antimicrobial activity of a new active food packaging containing cinnamon or oregano against *E. coli* and *S. aureus*. Anal. Bioanal. Chem..

[10] Vairappan CS, Nagappan T, Kulip J (2014). The essential oil profiles and antibacterial activity of six wild *Cinnamomum* species. Nat. Prod. Commun..

[11] Oliveira JDA (2014). Safety and tolerability of essential oil from *Cinnamomum zeylanicum* blume leaves with action on oral candidosis and its effect on the physical properties of the acrylic resin. Evid.-based Complement. Altern. Med..

[12] Ribeiro M, Malheiro J, Grenho L, Fernandes MH, Simões M (2018). Cytotoxicity and antimicrobial action of selected phytochemicals against planktonic and sessile *Streptococcus mutans*. PeerJ.

[13] de Oliveira Carvalho I (2020). In vitro anticariogenic and antibiofilm activities of toothpastes formulated with essential oils. Arch. Oral Biol..

[14] Isaac-Renton M, Li MK, Parsons LM (2015). Cinnamon spice and everything not nice: Many features of intraoral allergy to cinnamic aldehyde. Dermatitis.

[15] Olsen RV, Andersen HH, Møller HG, Eskelund PW, Arendt-Nielsen L (2014). Somatosensory and vasomotor manifestations of individual and combined stimulation of TRPM8 and TRPA1 using topical L-menthol and trans-cinnamaldehyde in healthy volunteers. Eur. J. Pain (United Kingdom).

[16] Wiwattanarattanabut K, Choonharuangdej S, Srithavaj T (2017). In vitro anti-cariogenic plaque effects of essential oils extracted from culinary herbs. J. Clin. Diagn. Res..

[17] Didry N, Dubreuil L, Pinkas M (1994). Activity of thymol, carvacrol, cinnamaldehyde and eugenol on oral bacteria. Pharm. Acta Helv..

[18] Chaudhari LKD (2012). Antimicrobial activity of commercially available essential oils against *Streptococcus mutans*. J. Contemp. Dent. Pract..

[19] Wang Y (2018). Antibacterial effects of cinnamon (*Cinnamomum zeylanicum*) bark essential oil on *Porphyromonas gingivalis*. Microb. Pathog..

[20] Yanakiev S (2020). Effects of cinnamon (*Cinnamomum* spp.) in dentistry: A review. Molecules.

[21] de Almeida LFD (2016). Efficacy of citronella and cinnamon essential oils on *Candida albicans* biofilms. Acta Odontol. Scand..

[22] Gupta D, Jain A (2015). Effect of cinnamon extract and chlorhexidine gluconate (0.2%) on the clinical level of dental plaque and gingival health: A 4-week, triple-blind randomized controlled trial. J. Int. Acad. Periodontol..

[23] Silva AF (2018). Cinnamaldehyde induces changes in the protein profile of *Salmonella* Typhimurium biofilm. Res. Microbiol..

[24] Wei QY, Xiong JJ, Jiang H, Zhang C, Wen Y (2011). The antimicrobial activities of the cinnamaldehyde adducts with amino acids. Int. J. Food Microbiol..

[25] Worreth S (2022). Cinnamaldehyde as antimicrobial in cellulose-based dental appliances. J. Appl. Microbiol..

[26] Li YQ, Kong DX, Wu H (2013). Analysis and evaluation of essential oil components of cinnamon barks using GC-MS and FTIR spectroscopy. Ind. Crops Prod..

[27] Paranagama PA (2001). A comparison of essential oil constituents of bark, leaf, root and fruit of cinnamon (*Cinnamomum zeylanicum* blum) grown in Sri Lanka. J. Natl. Sci. Found. Sri Lanka.

[28] He Z, Huang Z, Jiang W, Zhou W (2019). Antimicrobial activity of Cinnamaldehyde on *Streptococcus mutans* biofilms. Front. Microbiol..

[29] Goldbeck JC, do Nascimento JE, Jacob RG, Fiorentini ÂM, da Silva WP (2014). Bioactivity of essential oils from *Eucalyptus globulus* and *Eucalyptus urograndis* against planktonic cells and biofilms of *Streptococcus mutans*. Ind. Crops Prod..

[30] Sun Y (2018). Effects of sub-minimum inhibitory concentrations of lemon essential oil on the acid tolerance and biofilm formation of *Streptococcus mutans*. Arch. Oral Biol..

[31] Chung J (2018). Trans-cinnamic aldehyde inhibits *Aggregatibacter actinomycetemcomitans*-induced inflammation in THP-1–derived macrophages via autophagy activation. J. Periodontol..

[32] Iorgulescu G (2009). Saliva between normal and pathological. Important factors in determining systemic and oral health. J. Med. Life.

[33] Ojha PK (2022). Volatile constituent analysis of wintergreen essential oil and comparison with synthetic methyl salicylate for authentication. Plants.

[34] Abers M (2021). Antimicrobial activity of the volatile substances from essential oils. BMC Complement. Med. Ther..

[35] Ou Y (2022). Cinnamaldehyde protects against ligature-induced periodontitis through the inhibition of microbial accumulation and inflammatory responses of host immune cells. Food Funct..

[36] Hertan E, McCray J, Bankhead B, Kim KB (2022). Force profile assessment of direct-printed aligners versus thermoformed aligners and the effects of non-engaged surface patterns. Prog. Orthod..

[37] Gao L, Wichelhaus A (2017). Forces and moments delivered by the PET-G aligner to a maxillary central incisor for palatal tipping and intrusion. Angle Orthod..

[38] Kwon JS, Lee YK, Lim BS, Lim YK (2008). Force delivery properties of thermoplastic orthodontic materials. Am. J. Orthod. Dentofac. Orthop..

[39] Elkholy F, Schmidt S, Schmidt F, Amirkhani M, Lapatki BG (2021). Force decay of polyethylene terephthalate glycol aligner materials during simulation of typical clinical loading/unloading scenarios. J. Orofac. Orthop..

[40] Simon M, Keilig L, Schwarze J, Jung BA, Bourauel C (2014). Forces and moments generated by removable thermoplastic aligners: Incisor torque, premolar derotation, and molar distalization. Am. J. Orthod. Dentofac. Orthop..

[41] Dewhirst FE (2010). The human oral microbiome. J. Bacteriol..

[42] Lamont RJ, Koo H, Hajishengallis G (2018). The oral microbiota: Dynamic communities and host interactions. Nat. Rev. Microbiol..

[43] Kinane DF, Mombelli A (2012). Periodontal disease. Foreword. Front. Oral Biol..

[44] Zijnge V, Ammann T, Thurnheer T, Gmür R (2011). Subgingival biofilm structure. Periodontal Dis..

[45] Vielkind P, Jentsch H, Eschrich K, Rodloff AC, Stingu CS (2015). Prevalence of *Actinomyces* spp. in patients with chronic periodontitis. Int. J. Med. Microbiol..

[46] Zwietering MH, Jongenburger I, Rombouts FM, Van’t Riet K (1990). Modeling of the bacterial growth curve. Appl. Environ. Microbiol..

[47] R Core Team. R: A language and environment for statistical computing. *R Foundation for Statistical Computing* (2019).

[48] Kahm M, Hasenbrink G, Lichtenberg-Fraté H, Ludwig J, Kschischo M (2010). grofit: Fitting biological growth curves with *R*. J. Stat. Softw..

[49] Cho S-B (1995). Dependence of apatite formation on silica gel on its structure: Effect of heat treatment. J. Am. Ceram. Soc..

[50] Lombardo L (2017). Stress relaxation properties of four orthodontic aligner materials: A 24-hour in vitro study. Angle Orthod..

