# Supplementary material for: Antimicrobial and mechanical assessment of cellulose-based thermoformable material for invisible dental braces with natural essential oils protecting from biofilm formation
Source: Sci Rep. 2023 Aug 18;13:13428. doi: 10.1038/s41598-023-39320-1 (PMC10439145; doi:10.1038/s41598-023-39320-1)
Supplement: Supplementary file 1 — Supplementary Information. [file 41598_2023_39320_MOESM1_ESM.pdf]

SUPPLEMENTARY MATERIAL

Equipping invisible dental braces with synergistic natural antimicrobials protecting from biofilm formation.

Monika Astasov-Frauenhoffer[1], Livia Göldi[1], Nadja Rohr[1], Sarah Worreth[2], Elise Dard[3], Selina Hünerfauth[3], Tino Töpper[3], Jonas Zurflüh[4], Olivier Braissant[2*],

1) Department Research, University Center for Dental Medicine Basel UZB, University of Basel, Mattenstrasse 40, Basel, Switzerland
2) Center of Biomechanics and Biocalorimetry, c/o Department of Biomedical Engineering (DBE), University of Basel, Allschwil, Switzerland,
3) Bottmedical *AG* Technologiepark Basel Hochbergerstrasse 60c, 4057 Basel, Switzerland
4) Department Chemie, University of Basel, Mattenstrasse 24a, Basel, Switzerland

* corresponding author: Olivier.braissant@unibas.ch

Supplementary table S1: Essential oils and bioactive molecule considered in the first screening for antimicrobial effect against *Streptococcus mutans* and *S.mitis*

**Common name**	**IUPAC name**
Thymol	5-methyl-2-propan-2-yl-phenol
Carvacrol	2-methyl-5-propan-2-yl-phenol
Trans – Anethol	1-methoxy-4-[(*E*)-prop-1-enyl]benzene
Trans – Cinnamaldehyde	(*E*)-3-phenylprop-2-enal
L – Carvone	(5*R*)-2-methyl-5-prop-1-en-2-ylcyclohex-2-en-1-one
(+) – Catechine hydrate	(2*R*,3*S*)-2-(3,4-dihydroxyphenyl)-3,4-dihydro-2*H*-chromene-3,5,7-triol
Eucalyptol (1-8 cineole)	1,3,3-trimethyl-2-oxabicyclo[2.2.2]octane
Methyl salicilate	methyl 2-hydroxybenzoate
(R) – (+) – Limonene	(4*R*)-1-methyl-4-prop-1-en-2-ylcyclohexene
4 – Carvomenthenol	4-methyl-1-propan-2-ylcyclohex-3-en-1-ol
Eugenol	2-methoxy-4-prop-2-enylphenol
Linalol	3,7-dimethylocta-1,6-dien-3-ol
β - Citronellol	(3R)-3,7-dimethyloct-6-en-1-ol
Quercetin	2-(3,4-dihydroxyphenyl)-3,5,7-trihydroxychromen-4-one
Rutin hydrate (Quercetin-3-rutinoside hydrate)	2-(3,4-dihydroxyphenyl)-5,7-dihydroxy-3-[(2*S*,3*R*,4*S*,5*S*,6*R*)-3,4,5-trihydroxy-6-[[(2*R*,3*R*,4*R*,5*R*,6*S*)-3,4,5-trihydroxy-6-methyloxan-2-yl]oxymethyl]oxan-2-yl]oxychromen-4-one;hydrate
L – Menthol	(1*R*,2*S*,5*R*)-5-methyl-2-propan-2-ylcyclohexan-1-ol
DL – Camphor	(1*R*,4*R*)-1,7,7-trimethylbicyclo[2.2.1]heptan-2-one
Spearmint oil (rectified)	Sigma Aldrich reference W303208
Citronellol	3,7-dimethyloct-6-en-1-ol
(+) – Citronellal	(3*R*)-3,7-dimethyloct-6-enal

Supplementary table S2: Concentration of bioactive molecule in the aligner material with increasing loading time. See main text for procedure and measurement accuracy.

loading time	Cinnamaldehyde	Limonene	Trans-anethole	Methyl Salicylate	Eucalyptol	sum
[min]	[mg·cm^{-2}]	[mg·cm^{-2}]	[mg·cm^{-2}]	[mg·cm^{-2}]	[mg·cm^{-2}]	[mg·cm^{-2}]
10	0.086	0.008	0.030	0.076	0.009	0.210
30	0.087	0.018	0.042	0.095	0.013	0.256
60	0.254	0.015	0.068	0.180	0.027	0.544
360	0.367	0.000	0.076	0.217	0.020	0.680

Supplementary table S3: Concentration of bioactive molecule remaining in the aligner material with increasing rinsing time. All measurments were performed with samples loaded for 1 hour. See main text for procedure and measurement accuracy.

loading time	Cinnamaldehyde	Limonene	Trans-anethole	Methyl Salicylate	Eucalyptol	sum
[min]	[mg·cm^{-2}]	[mg·cm^{-2}]	[mg·cm^{-2}]	[mg·cm^{-2}]	[mg·cm^{-2}]	[mg·cm^{-2}]
1	0.218	0.015	0.042	0.124	0.023	0.422
4	0.028	0.000	0.017	0.029	0.003	0.076
6	0.009	0.000	0.006	0.010	0.001	0.026
12	0.000	0.000	0.004	0.008	0.000	0.012
24	0.000	0.000	0.000	0.008	0.000	0.009

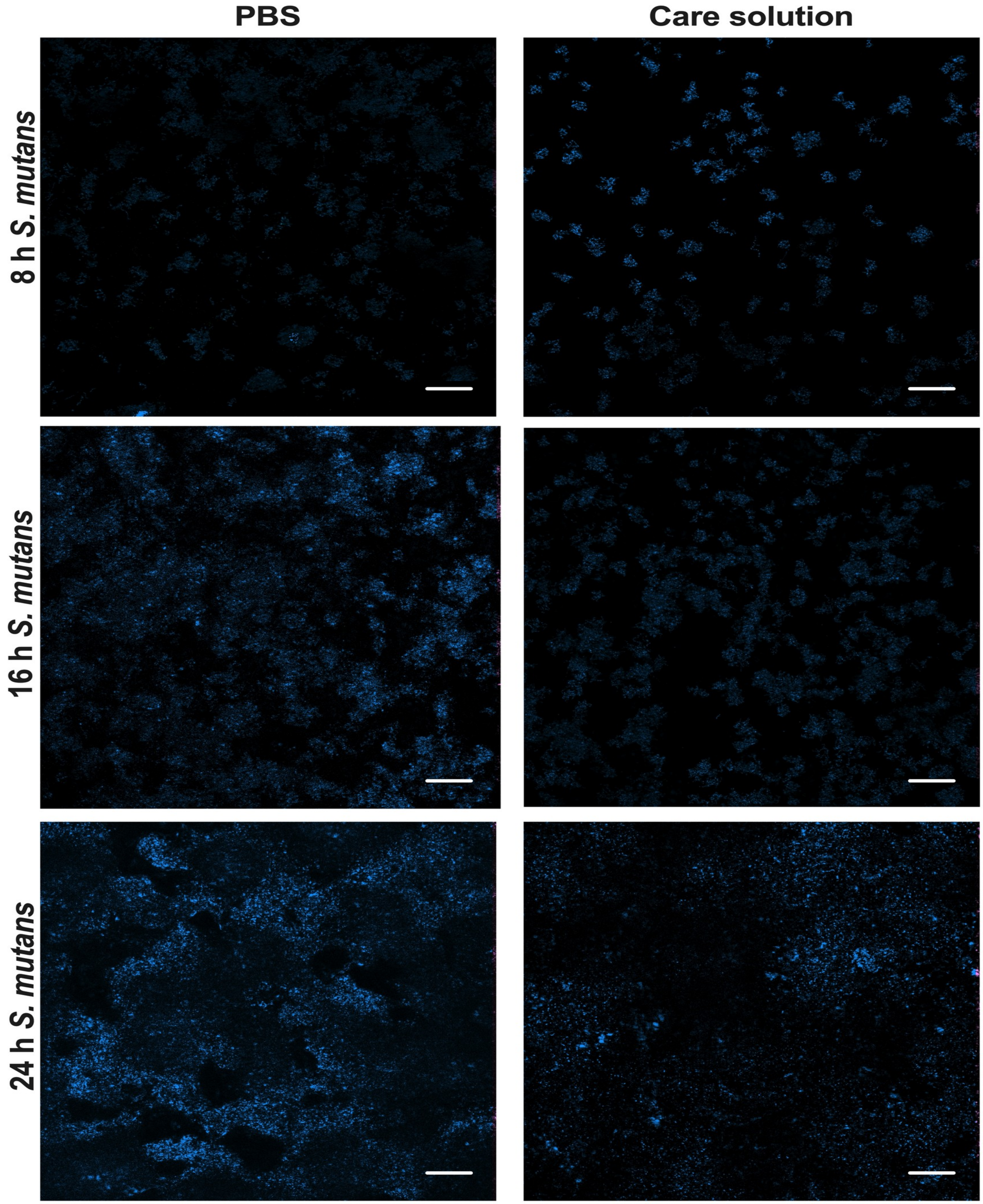

Figure S4: Time series showing evolution of biofilm formation by *S. mutans* on unloaded NA1.750 (exposed to PBS) and NA1.750 loaded with the care solution containing 5 essential oils (see main text for details) at 8, 16 and 24 hours. Cells were stained with DAPI, scale bar is 20 µm

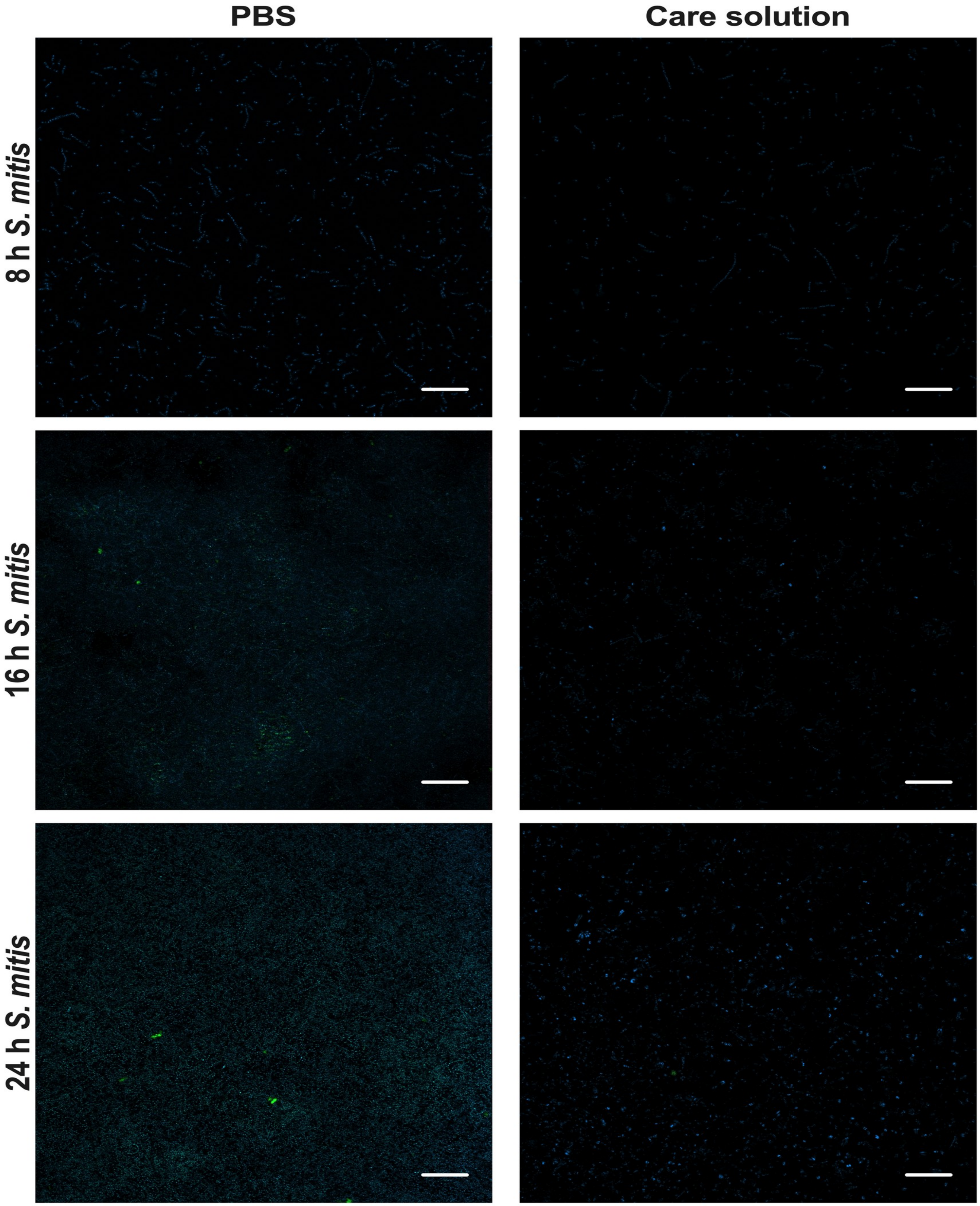

Figure S5: Time series showing evolution of biofilm formation by *S. mutis* on unloaded NA1.750 (exposed to PBS) and NA1.750 loaded with the care solution containing 5 essential oils (see main text for details) at 8, 16 and 24 hours. Cells were stained with DAPI, scale bar is 20 μm.
